# Differential MicroRNA Regulation Correlates with Alternative Polyadenylation Pattern between Breast Cancer and Normal Cells

**DOI:** 10.1371/journal.pone.0056958

**Published:** 2013-02-21

**Authors:** Hao-Han Liaw, Chen-Ching Lin, Hsueh-Fen Juan, Hsuan-Cheng Huang

**Affiliations:** 1 Institute of Biomedical Informatics, Center for Systems and Synthetic Biology, National Yang-Ming University, Taipei, Taiwan; 2 Department of Life Science, Graduate Institute of Biomedical Electronics and Bioinformatics, National Taiwan University, Taipei, Taiwan; The John Curtin School of Medical Research, Australia

## Abstract

Alternative polyadenylation (APA) could result in mRNA isoforms with variable lengths of 3′ UTRs. Gain of microRNA target sites in the 3′ UTR of a long mRNA isoform may cause different regulation from the corresponding short isoform. It has been known that cancer cells globally exhibit a lower ratio of long and short isoforms (LSR); that is, they tend to express larger amounts of short isoforms. The objective of this study is to illustrate the relationship between microRNA differential regulation and LSR. We retrieved public APA annotations and isoform expression profiles of breast cancer and normal cells from a high-throughput sequencing method study specific for the mRNA 3′ end. Combining microRNA expression profiles, we performed statistical analysis to reveal and estimate microRNA regulation on APA patterns in a global scale. First, we found that the amount of microRNA target sites in the alternative UTR (aUTR), the region only present in long isoforms, could affect the LSR of the target genes. Second, we observed that the genes whose aUTRs were targeted by up-regulated microRNAs in cancer cells had an overall lower LSR. Furthermore, the target sites of up-regulated microRNAs tended to appear in aUTRs. Finally, we demonstrated that the amount of target sites for up-regulated microRNAs in aUTRs correlated with the LSR change between cancer and normal cells. The results indicate that up-regulation of microRNAs might cause lower LSRs of target genes in cancer cells through degradation of their long isoforms. Our findings provide evidence of how microRNAs might play a crucial role in APA pattern shifts from normal to cancerous or proliferative states.

## Introduction

MicroRNAs have been shown to be a very important post-transcriptional regulator of gene expression. MicroRNAs regulate gene expression by complementarily binding to recognition sequences, mostly 6–8 nt, in the 3′ untranslated regions (3′ UTR) of their target mRNAs, thus inducing mRNA degradation and/or blocking mRNA translation. They have been found to control a wide range of biological processes including development, differentiation, growth, apoptosis, and proliferation [1]–[5]. Moreover, several microRNAs have been shown to be expressed abnormally in many cancer types [6], indicating that microRNA is closely related to carcinogenesis.

Cleavage and polyadenylation are RNA maturation events that cut and add an oligo(dA) tail to the 3′ end of the nascent transcript [7], [8]. This processing is to prevent mRNAs from degradation and to increase their stability. Previous studies have indicated that more than half of human genes possess multiple polyadenylation sites [9], called *alternative polyadenylation* (APA), which may produce mRNA isoforms with different protein-coding regions or 3′ UTRs of variable length (when APA occurs in the last exon). The intuitive impact of the latter case is gain of some cis-regulatory elements, such as microRNA target sites, in the 3′ UTR of the long isoform, which may cause different regulation from the short isoform. It has been known that the expression ratio between long isoforms and short isoforms (LSR) of overall genes is variable across different tissues or cell types [10], [11]. Interestingly, Sandberg et al. found that the ratio has a strong negative correlation with cell proliferative state [12]. In addition, cancer cells exhibit significantly lower ratios than normal tissues and untransformed cells [13].

**Figure 1:**
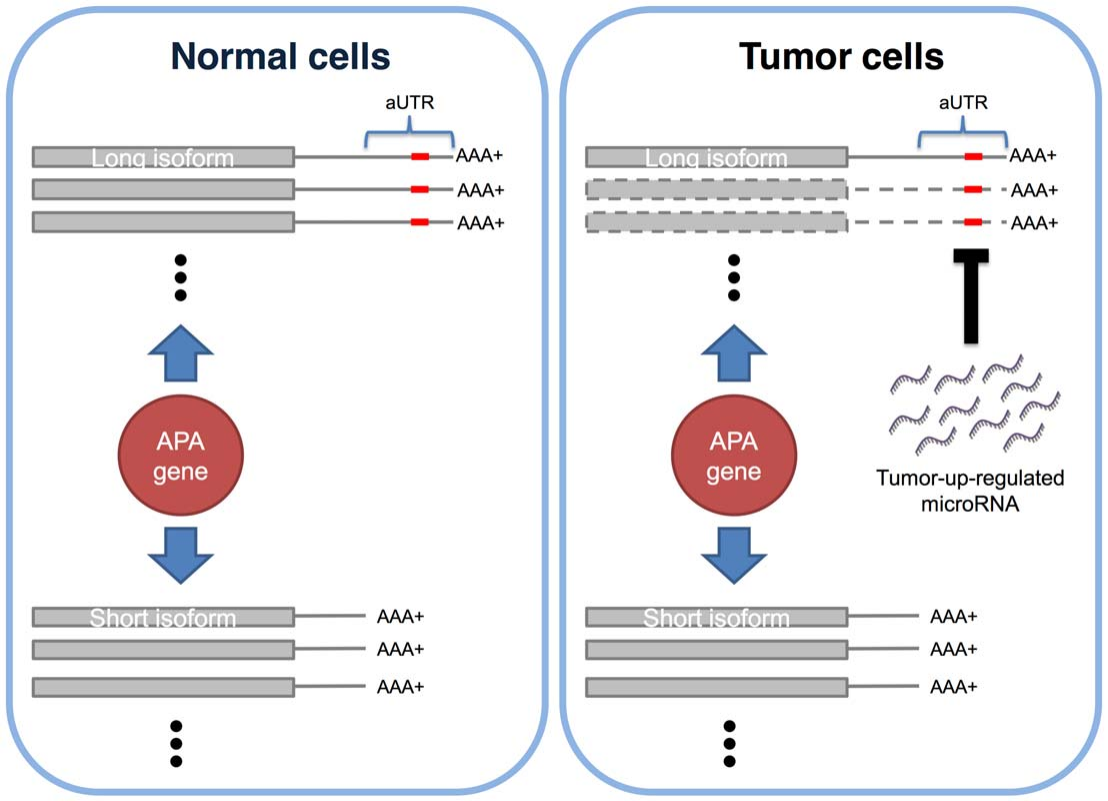
Hypothesis of microRNA regulation on alternative polyadenylation. Here, we illustrate an example of our hypothesis. Left and right panels represent the situations under normal and tumor cells, respectively. Initially, the APA gene produces long and short isoforms in both normal and tumor cells. However, due to modulation of tumor-up-regulated microRNA on the aUTR, the long isoforms in tumor cells are greatly degraded (transcripts with dashed outlines). The expression ratio between long isoforms and short isoforms (LSR) is thereby lower in tumor than normal cells.

The observed lower LSR may result from two possible mechanisms: (1) the production rate of the short isoform is higher than that of the long isoform because of some pressure; (2) the alternative UTR (aUTR), the region that presents in long but not short isoforms, confers a higher degradation rate on long isoforms. The former one has been demonstrated, showing that expression of several 3′ processing factors [13]–[15] and transcriptional activity of the APA gene itself [16] both elevated the production rate of short isoforms over long isoforms. The latter mechanism has also been examined, and findings suggested that some microRNAs repressed the long isoforms of their target genes in the tissue where they were specifically expressed, such as miR-1 and miR-124a [17]. In cancer cells, it was found that the microRNA target sites in aUTRs speed up the degradation rate of long isoforms [13] for some sample microRNAs and a small set of target genes. However, there has not yet been a global analysis on how the expression levels of microRNAs and their target sites in the aUTRs influence the LSR of target genes in cancer versus normal cells. Here, we combined and analyzed published APA annotation and mRNA isoform expression information [18], and microRNA expression profiles [19] of breast cancer and normal cells. Our aim is to verify the hypothesis that some abnormally up-regulated microRNAs in breast cancer cells bind to the aUTRs of their target genes, resulting in a higher degradation rate of the long isoforms, and thus the observed overall LSR of these target genes will tend to be lower than in normal cells (Figure 1).

## Results

### Preference of APA Genes for Short Isoforms in Breast Cancer Cells

**Figure 2:**
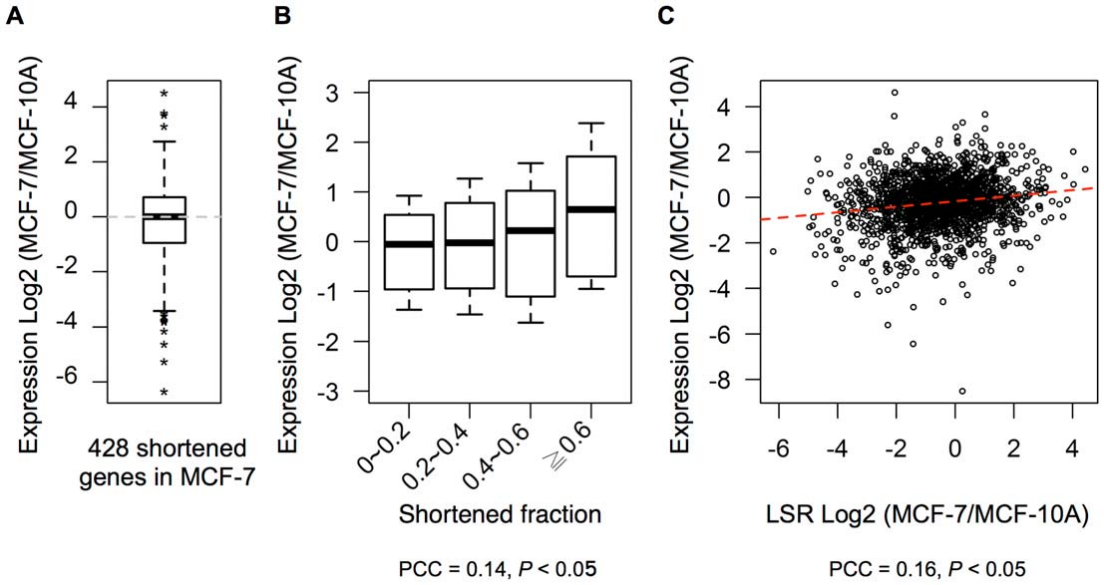
Correlation among expression level, shortened fraction and LSR. (A) Box plot for log2 ratios of mRNA expression levels between MCF-7 and MCF-10A cells of the shortened genes reported by Fu et al., 2011. Expression level is the total read count of all isoforms for each gene. (B) Box plots for log2 ratios of mRNA expression levels between MCF-7 and MCF-10A cells against shortened fraction in MCF-7 cells. For each gene, shortened fraction is calculated by dividing average shifted length in MCF-7 by weighted 3′ UTR length in MCF-10A. All data originate from Fu et al., 2011. (C) Scatter plot for mRNA expression level versus LSR between MCF-7 and MCF-10A cells of all APA genes (*y*-axis: log2 ratio of mRNA expression level between MCF-7 and MCF-10A cells; *x*-axis: log2 ratio of LSR, where the lower the value, the higher the proportion of short isoform in MCF-7 versus MCF-10A). A positive correlation can be observed. The red dashed line denotes the linear regression line.

First of all, we retrieved APA site annotation and isoform expression data from a novel and high-throughput sequencing method study specific for the mRNA 3′ end [18]. The original study found 489 genes with a significant difference in APA pattern between MCF-7 and MCF-10A, and 88% (428/489) of them were observed to have a higher proportion of short isoforms in MCF-7 than in MCF-10A (namely, their 3′ UTRs were “shortened” in MCF-7). According to the mechanism of differential production, if the increase in transcription rate of short isoform was the major cause of the APA pattern shift, the expression levels of these APA genes would increase in MCF-7 because the short isoform could escape microRNA regulation and gain a longer mRNA half-life [13]. However, an overall expression change between MCF-7 and MCF-10A for these shortened genes was not observed (Figure 2A). We further reasoned that the shortened fraction of 3′ UTR (that is, dividing reduced length in MCF-7 by 3′ UTR length in MCF-10A) might influence expression levels more directly. If the short isoform and long isoform of an APA gene possessed similar 3′ UTR length, then their mRNA properties would be substantially the same. Although its APA pattern would be shifted to express more short isoform in MCF-7, the consequence of this would not be obvious. As expected, we found that the expression levels of shortened genes increased in MCF-7 while their shortened fraction became larger (Figure 2B). This implied that these genes might increase expression levels through escaping the regulation of microRNA and other destabilizing elements, consistent with the effects of differential production. However, when considering all APA genes, there was no negative correlation between LSR change and expression change (Figure 2C), implying that some underlying mechanisms other than differential production might play an important role in global APA pattern shifts. Previous APA studies mainly focused on the mechanism of differential production – for instance, influence of 3′ processing factors and transcriptional activity on APA pattern – while some examined just a few individual microRNAs and target genes for the mechanism of differential degradation. How extensive and effective the latter is involved in global APA pattern shifts has not been delineated yet. In this study, we used the latest high-throughput data on APA genes to illuminate microRNA modulation on APA pattern.

### MicroRNA Target Site Versus LSR

**Figure 3:**
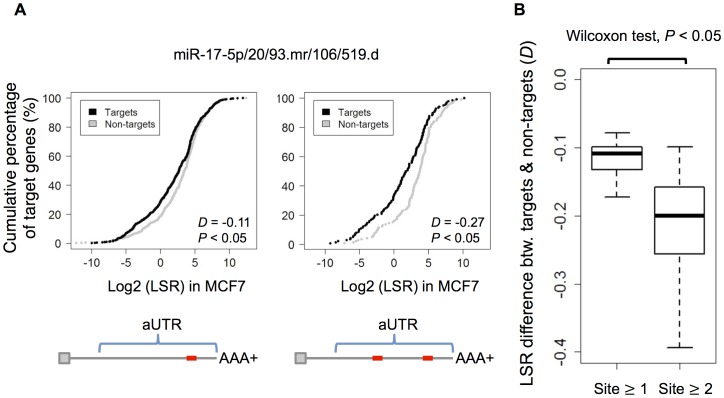
MicroRNAs are associated with LSR of their target gene sets. (A) Cumulative density plot of LSR for target genes (black) and non-target genes (gray) of the miR-17-5p/20/93.mr/106/519.d family as an example. The KS-test *D* value reflects the LSR difference between target and non-target genes. A negative *D* value means that the overall LSR of target genes is lower. The left figure consists of the target genes with one or more target sites in aUTR, while the right figure consists of those with two or more sites. (B) Box plot for *D* values of the microRNAs whose target gene LSRs decrease significantly (*P*<0.05, KS-test, 101 out of 130 total microRNAs) (left). The *D* values of these microRNAs decrease further if two or more target sites are required (right).

Assuming that a gene initially produces long and short isoforms with similar LSR between cancer and normal cells, and the microRNAs whose target sites are located in the aUTR are up-regulated in cancer cells, the long isoforms in cancer cells would preferentially be degraded, resulting in a lower LSR than observed in normal cells. Although global microRNA down-regulation is a common trait of human cancer, spike-in calibrations of microRNA sequencing data indicated that there was no significant change in the overall microRNA content between breast cancer and normal samples; furthermore, the overall microRNA content in MCF-7 was about 4.7 times greater than in MCF-10A [19]. To verify the aforementioned hypothesis, first we simply examined whether or not the microRNA target sites in an aUTR affected the LSR of the genes. Hence, comparisons of overall LSR between target genes and non-target genes of each microRNA were performed. For each microRNA, we retrieved the target gene set that possessed its target site(s) in the aUTR. Then, a non-target set of the same size was randomly sampled from the genes without its target site(s) in the aUTR, but keeping gene expression and 3′ UTR length similar to target genes to minimize the influence of other factors on LSR. We assumed that the only difference between the target and non-target genes here was whether the target site(s) of the microRNA presented in the aUTR or not, so we could estimate the microRNA influence on LSR between these two sets. In MCF-7, we found that the target gene sets of 78% (101/130) of microRNA families exhibited significant (*P*<0.05, KS-test) overall lower LSRs than non-target gene sets. Moreover, if we further restricted the criterion for target genes to that the target sites in the aUTR must be more than 1, the overall LSR of target gene sets became significantly much lower than with the original criterion (*P*<0.05, Wilcoxon-test; Figure 3). The same analysis on MCF-10A obtained similar results to those observed in MCF-7. This clear dosage effect of microRNA regulation on aUTR gives us confidence to reason that the presence of microRNA target sites in aUTRs result in degradation of long isoforms.

### MicroRNA Differential Expression Versus LSR Change of Targets

**Figure 4:**
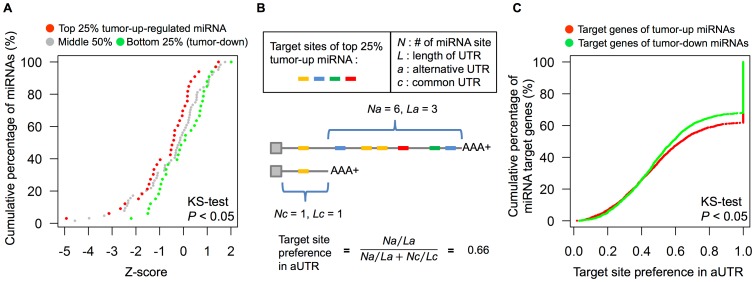
MicroRNA differential expression versus LSR change of targets and preference of target sites in aUTRs. (A) Cumulative density plot of LSR change for target genes of each microRNA. *Z*-score represents an estimator of target gene LSR change compared to background (see Materials and Methods). A negative *z*-score means that the overall LSR is lower in MCF-7 cells. Target genes of up-regulated microRNAs (red) show a significantly decreased LSR in MCF-7 versus the down-regulated ones (green). (B) An illustration for estimating the preference of microRNA target sites to appear in aUTRs. A higher value means that the target sites are more likely to appear in the aUTR of the APA gene. (C) Cumulative density plot of the preference of microRNA target sites to appear in aUTRs. Each point represents an aUTR target gene with the preference value exemplified in (B). MCF-7-up-regulated microRNA target sites (red) exhibit a significantly higher preference than MCF-7-down-regulated microRNA target sites (green).

To further clarify if there were some microRNAs up-regulated in MCF-7, thus resulting in their target gene sets exhibiting lower LSR than observed in MCF-10A, we calculated the fold-change of each microRNA family and estimated the change in overall LSR of its target gene set between MCF-7 and MCF-10A. We then binned the target gene sets into 3 groups by fold-change of their targeting microRNAs: (1) top 25% (up-regulated microRNAs in MCF-7), (2) middle 50%, and (3) bottom 25% (down-regulated in MCF-7). As expected, we found that globally the target gene sets of Group 1 showed a significantly decreased LSR from MCF-10 to MCF-7 versus the LSR of Group 3 (*P*<0.05, KS-test), and the extent of Group 2 was exactly between them (Figure 4A). Furthermore, four microRNA families in Group 1 with significant LSR decreases of their target genes (z<−2 compared to background) were selected to survey prior literature. Interestingly, we found that two of these microRNA families have been reported to be associated with a mechanism by which microRNAs are up-regulated, and thus their aUTR target genes exhibited overall lower LSRs (miR-421 in B-cell lymphoma and miR-25/32/92/92ab/363/367 in activated T-cells) [12], [20]. These results indicated that microRNAs with differential expression between normal and cancer cells might affect the overall LSR of their aUTR target genes. Consistent with our hypothesis, some up-regulated microRNAs seemed to strongly degrade long isoforms of their aUTR target genes and thus result in a lower overall LSR.

### Preference of microRNA Target Sites to Appear in aUTRs

If long isoforms of some APA genes were destined to be degraded by up-regulated microRNA under particular conditions (such as high-proliferative state or cancerous state) for some important biological functions, then evolutionarily the target sites of these up-regulated microRNAs would be retained in aUTRs to achieve the purpose more effectively. To test this assumption, we estimated the preference of target sites in aUTRs for the microRNA groups aforementioned. It was observed that the target sites of up-regulated microRNAs in MCF-7 cells had a higher preference for appearing in aUTRs than those of down-regulated ones (*P*<0.05, KS-test; Figures 4B and 4C). Summarizing this result and the previous section, we conclude that not only the up-regulation of microRNAs but also their higher target-site preference for appearing in aUTRs might result in the lower overall LSR of their aUTR target genes.

### Target Sites of Up-regulated microRNAs Versus LSR Change in MCF-7 Cells

**Figure 5:**
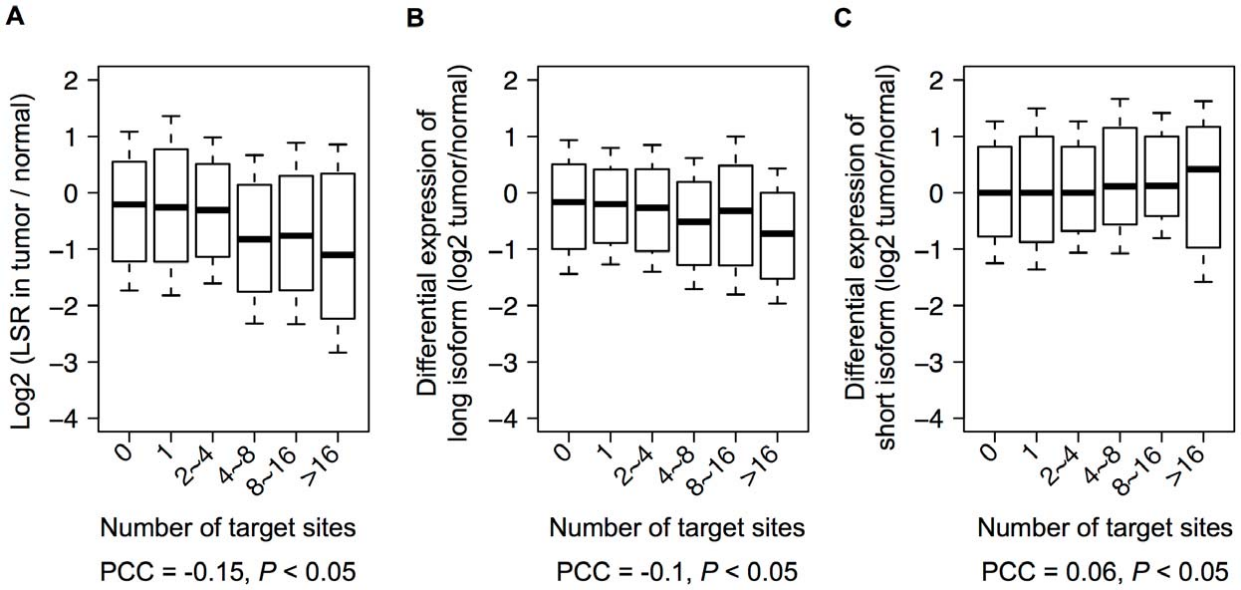
Correlation between target site abundance of up-regulated microRNAs and LSR change in MCF-7 cells. Box plots for log2 ratios of (A) LSR, (B) long-isoform expression, and (C) short-isoform expression change between MCF-7 and MCF-10A cells against target-site abundance of MCF-7-up-regulated microRNAs in aUTRs. The genes with more target sites encounter a greater LSR decrease and long-isoform expression decline in MCF-7 cells. However, differential expression of the short isoform shows little correlation with target site abundance.

At the beginning, we observed a dosage effect of microRNA target sites on LSR. Then, we found that abnormally up-regulated microRNAs in MCF-7 would cause lower LSRs of their aUTR target genes. In addition, we observed that target sites of these microRNAs preferentially appeared in aUTR. Accordingly, we reasoned that target site number of these MCF-7-up-regulated microRNAs in aUTR would affect LSR change between MCF-7 and MCF-10A. In other words, long isoforms of the genes are effectively degraded in MCF-7 as target site number rises; thus, LSR change between MCF-7 and MCF-10A should became larger. As expected, the results show a significant negative correlation between LSR change (defined as the log2 ratio of LSR between MCF-7 and MCF-10A) and target site abundance of MCF-7-up-regulated microRNAs in aUTRs (*P*<0.05, Pearson's *r* = −0.15; Figure 5A). Furthermore, when we tested for correlations between long isoform change and target site number, a similar result was obtained (*P*<0.05, *r* = −0.10; Figure 5B) that was consistent with LSR change; however, the outcome of short isoform change was not (*P*<0.05, *r* = 0.06; Figure 5C). These findings indicated that the lower LSR in MCF-7 we observed was indeed caused by degradation of long isoforms. On the other hand, we found that 27% of APA genes were not targeted by these MCF-7-up-regulated microRNAs at aUTRs, and the LSR changes of this group between MCF-7 and MCF-10A were the least (Figure 5A, the leftmost box plot), although their LSR still decreased slightly in MCF-7. Moreover, those shortened genes reported by Fu et al. [18] were enriched in the other 73% of APA genes whose aUTR were targeted by MCF-7-up-regulated microRNAs (215 vs. 185 expected by chance; Fisher’s exact test, *P*<0.05). These results suggested that the phenomenon of differential degradation caused by microRNAs might enhance global LSR decreases in cancer cells.

## Discussion

We provide here an in-depth investigation into APA events that differ between breast cancer and normal cells. It has been known that overall APA patterns in cancer cells tend to express more short isoforms than long isoforms [13], but the underlying causes were not yet fully understood. Thus, we aimed to analyze and reveal the influence of microRNA regulation on APA pattern. It is expected that the microRNA target sites only present in aUTR may result in higher degradation rate of long isoforms than that of short isoforms. To demonstrate this, we first showed that aUTR target genes of microRNAs had lower proportions of long isoforms than non-target ones. Moreover, the genes that possessed two or more target sites in aUTRs had even lower proportions of long isoforms: that is, a dosage effect was observed. In addition to target-site abundance in aUTRs, the microRNA expression level could also amplify the effect of regulation. We found that the aUTR target genes of abnormally up-regulated microRNAs in cancer lose larger proportions of long isoforms. In addition, the target sites of these microRNAs preferentially appeared in aUTRs. It seemed that evolutionarily the microRNAs tended to degrade long isoforms of their target genes in specific conditions like proliferative or cancerous states. The consequence of this mechanism may have important biological significance. For instance, previous research on satellite cells, or adult muscle stem cells, found that different expression levels of miR-206 regulating the aUTR of Pax3 could influence cell proliferation and differentiation [21]. In our case, we found that miR-25/32/92/92ab/363/367 was abnormally up-regulated in MCF-7 and the overall LSR of its aUTR target genes substantially decreased from MCF-10A to MCF-7. One of the mature microRNAs in this family, miR-92, belongs to a notable proliferative and anti-apoptotic microRNA cluster, miR-17-92 cluster [22]. Whether its effect on the APA patterns of its target genes directly enhances cell proliferation of MCF-7 needs further investigation. Finally, we showed that LSR decreases in aUTR target genes of these abnormally up-regulated microRNAs were associated with target site number in a dose-dependent fashion, and through this demonstrated the major cause of LSR decreases was indeed degradation of long isoforms.

The APA pattern information used throughout the study was retrieved from a novel and high-throughput sequencing method study specific for the mRNA 3′ end [18]. It is overwhelmingly superior to previous resources used for APA studies: e.g., EST/cDNA data with its high false-positive rate, microarray data with its low resolution, and RNA-seq data that use EST-inferred APA sites as mapping reference. Combining this elegant data and microRNA sequencing data [19], we revealed and estimated microRNA regulation on APA patterns on a global scale. The results were plausible and consistent with characteristics of microRNA and previous studies. More serial sequencing data are required to further investigate whether tumor-related microRNAs and their modulation on APA pattern are associated with tumor progression. On the other hand, development of 3′ end sequencing technology rise up rapidly in recent years and has been applied to various model organisms such as yeast, *C. elegans*, zebrafish and mammals [23]–[28]. These mRNA 3′ end maps provide valuable resources for APA related studies. Combining them with microRNA expression profiles from the corresponding species and tissues will allow us to further clarify the ins and outs of microRNA regulation on APA pattern through their evolutionary relationship.

In addition to microRNA regulation on APA pattern, expression level of 3′ processing factors was associated with APA pattern [14]: that is, overall APA genes tended to express short isoforms while these factors were up-regulated. It was observed that the expression of several 3′-processing factors, including CPSF1 and CSTF2, were overall higher in breast cancer than normal cell lines [13], so this may be another important factor influencing APA pattern differences between MCF-7 and MCF-10A. Also, it has been proposed that transcriptional activity affects the LSR of APA genes at the transcription level. Ji et al. [16] found that globally short 3′UTR isoforms were relatively more abundant when genes were highly expressed whereas long 3′UTR isoforms were relatively more abundant when genes were lowly expressed. While the transcriptional activity might set the global trend of APA pattern, differentially expressed microRNAs acting on aUTRs could further the pattern shift of their target genes. It is known that transcription factors (TF) play an important role in controlling transcriptional activity, and they have been identified as having significant co-regulation on target genes with microRNAs [29]. Thus it will be interesting and promising to further investigate if combining the influences of TFs and microRNAs could explain the global lower LSR observed in proliferative or cancer cells more completely.

In this study, we have revealed microRNA regulation on APA pattern in a global scale. We proposed that, through abnormal up-regulation of expression levels and preferential evolutionary retention of their target sequences in aUTRs, some microRNAs in breast cancer might greatly degrade the long mRNA isoforms of their target genes, and thus result in a significant decrease of LSR. Our findings provide evidence of how microRNAs might play a crucial role in APA pattern shifts from normal to cancerous or proliferative states.

## Materials and Methods

### APA Annotation and mRNA Isoform Expression Data

The APA annotation and isoform expression data were collected from a sequencing method study that pinpointed APA sites and quantified expression levels by directly sequencing poly(A) tails of transcripts in MCF-7 and MCF-10A cell lines [18]. MCF-7 cells are broadly used as a model of human breast cancer, while MCF-10A cells come from a non-tumorigenic epithelial cell line that is usually used as a normal control for breast cancer research. Because a gene may possess more than two APA sites, for simplicity, we defined the transcript that used the 5′ most APA site as the short isoform of the gene, while the transcript that used the 3′ most APA site as the long isoform of the gene. And we defined the section between these two sites as the aUTR of the long isoform relative to the short isoform. In addition, if the read count of either the short or long isoform was zero or if the total read counts of both isoforms were less than 15, the genes were filtered out for noise reduction.

### MicroRNA Expression Profiles

The expression profiles of microRNAs in MCF-7 and MCF-10A were collected from a microRNA deep sequencing data [19]. The data provided read counts of each mature microRNA that was discernible. Because different mature microRNAs may have identical seed regions and thus target the same binding sites, we grouped mature microRNAs into families according to microRNA family information from the TargetScan database (release 5.2) [30]. MicroRNA families which did not target any aUTR were filtered out. A total of 130 microRNA families were discernible and collected.

### MicroRNA Target Sites

We collected and used the predicted conserved target sites of conserved microRNA families from the TargetScan database [30] to perform analysis.

### Overall LSR of Targets Versus Non-targets

For estimating the overall LSR of aUTR target genes versus non-target genes, first we randomly chose non-target genes while closely matching their mRNA expression levels and 3′ UTR lengths to target genes. And for each microRNA family we drew a cumulative density plot of LSR with two lines representing target and non-target genes. Finally, we performed a Kolmogorov–Smirnov test (KS-test) to estimate the difference between these two lines. The *D* value of the outcome of the KS-test could be regarded as the extent of difference. A lower negative value indicates a lower overall LSR of target genes versus non-target genes. A *p*-value lower than 0.05 was regarded as significant difference. A schema of the above descriptions is shown in Figure S1.

### MicroRNA Differential Expression

To estimate differential expression of microRNA families, we first ranked them by their expression levels in MCF-7 and MCF-10A. And for each microRNA, we divided its rank in MCF-7 by the one in MCF-10A, calculating its rank-fold-change. We further binned the microRNAs into three groups: (1) top 25% (MCF-7-up-regulated), (2) middle 50%, and (3) bottom 25% (MCF-7-down-regulated), according to fold-change.

### LSR Change of Targets between Cell Types

Because there was a background bias of the genes in MCF-7 globally exhibiting lower LSR than in MCF-10A, the LSR change between MCF-7 and MCF-10A of microRNA target genes was compared to the LSR change of non-target genes. For each microRNA, the *D* value of its target genes between MCF-7 and MCF-10A was calculated. And, we randomly chose non-target genes 100 times, and each time we calculated the *D* value of these genes in between MCF-7 and MCF-10A. A normal distribution of *D* values was then formed by these 100 non-target gene sets. Next, the *z*-score of the *D* value of the target gene set was calculated. This *z*-score was regarded as the estimator of the LSR change of target genes versus background: a lower negative value means that the overall LSR of target genes is statistically lower in MCF-7. The above description is illustrated in Figure S2.

### Target-site Preference in aUTR

The preference of target sites in the aUTR of each gene was estimated for the top and bottom of differentially expressed microRNA-family groups, respectively. For each group, we defined a preference score for each aUTR target gene of these microRNAs as calculated by the following formula:
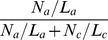



*N_a_* is the number of target sites in the aUTR; *N_c_* is the number of target sites in the common UTR (cUTR), the region both short and long isoforms possess; *L_a_* and *L_c_* are the lengths of aUTR and cUTR, respectively. Then we drew the cumulative density plots of preference scores for these two target gene sets. Finally, we performed KS-tests to estimate their differences. The above description is illustrated in Figure 4B.

## Supporting Information

Figures S1 **An illustration for estimating overall LSR of targets versus non-targets.** For each microRNA, we can draw a cumulative density plot (bottom). The example plot means 80% of the target genes, but only 30% of non-target genes, express more short isoforms than long isoforms. This indicated that the overall LSR is lower in target genes.
(TIFF)

Figure S2 **An illustration for estimating overall LSR change of microRNA target genes between MCF-7 and MCF-10A.** For each microRNA, the *D* value of its target genes between MCF-7 and MCF-10A was calculated. And we randomly chose non-target genes 100 times, and each time we calculated the *D* value of these genes between MCF-7 and MCF-10A. A normal distribution of *D* values was then formed by these 100 non-target gene sets. Next, the *z*-score of the *D* value of the target gene set was calculated. This *z*-score was regarded as the estimator of the LSR change of target genes compared to background, a lower negative value meaning that the overall LSR of target genes was statistically lower in MCF-7.
(TIFF)
