# Supplementary figures and images for: Differential MicroRNA Regulation Correlates with Alternative Polyadenylation Pattern between Breast Cancer and Normal Cells
Source: PLoS One. 2013 Feb 21;8(2):e56958. doi: 10.1371/journal.pone.0056958 (PMC3578872; doi:10.1371/journal.pone.0056958)

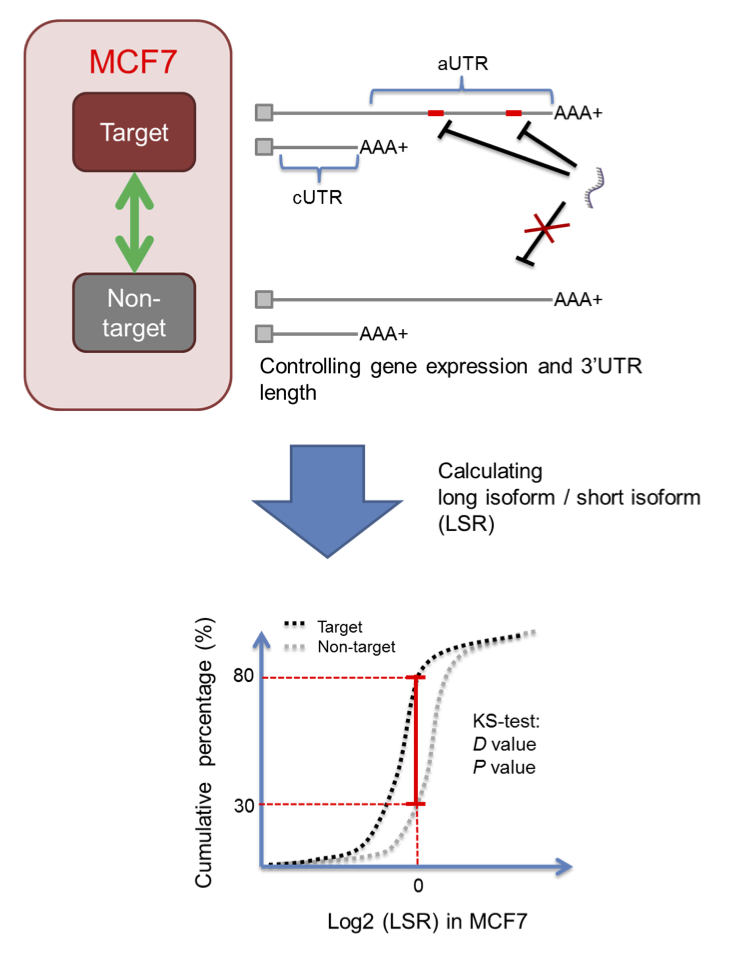

Supplement: Figures S1 — An illustration for estimating overall LSR of targets versus non-targets. For each microRNA, we can draw a cumulative density plot (bottom). The example plot means 80% of the target genes, but only 30% of non-target genes, express more short isoforms than long isoforms. This indicated that the overall LSR is lower in target genes. (TIFF) [file pone.0056958.s001.tiff]

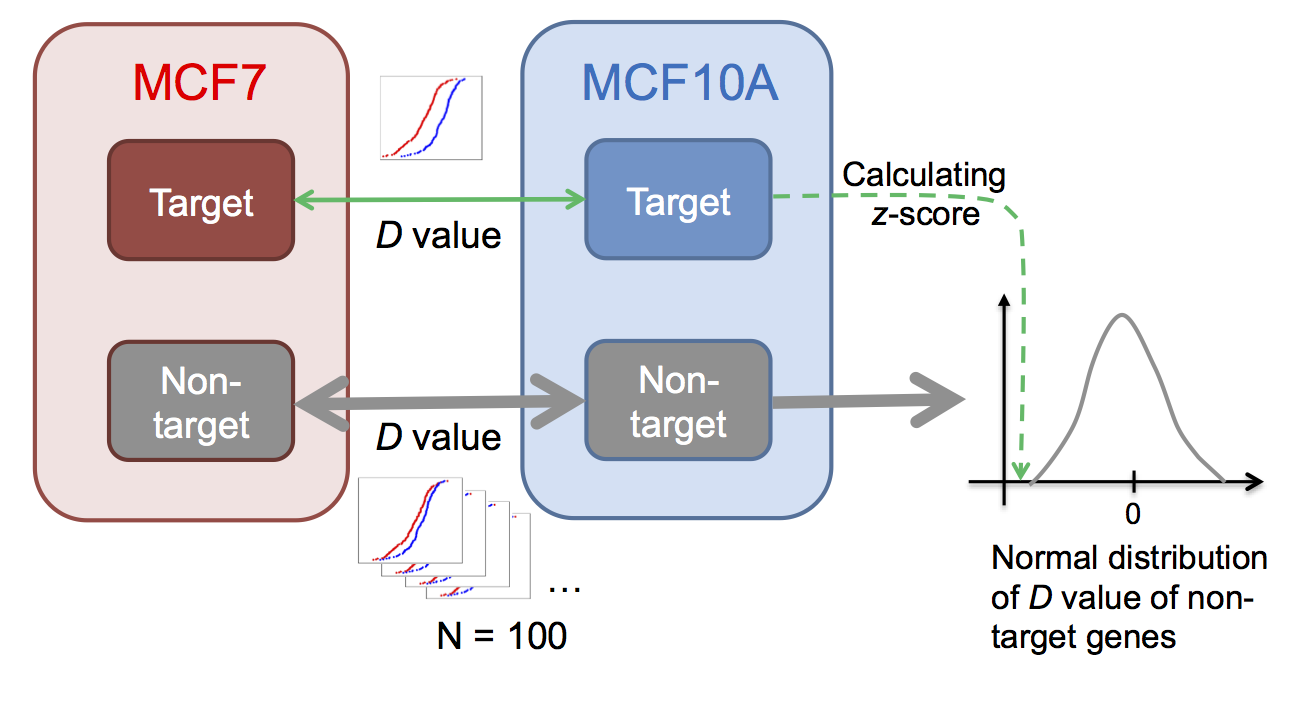

Supplement: Figure S2 — An illustration for estimating overall LSR change of microRNA target genes between MCF-7 and MCF-10A. For each microRNA, the D value of its target genes between MCF-7 and MCF-10A was calculated. And we randomly chose non-target genes 100 times, and each time we calculated the D value of these genes between MCF-7 and MCF-10A. A normal distribution of D values was then formed by these 100 non-target gene sets. Next, the z-score of the D value of the target gene set was calculated. This z-score was regarded as the estimator of the LSR change of target genes compared to background, a lower negative value meaning that the overall LSR of target genes was statistically lower in MCF-7. (TIFF) [file pone.0056958.s002.tiff]
